# Facile Preparation of Porous Microfiber from Poly-3-(R)-Hydroxybutyrate and Its Application

**DOI:** 10.3390/ma13010086

**Published:** 2019-12-23

**Authors:** Vojtech Kundrat, Petra Matouskova, Ivana Marova

**Affiliations:** Department of Food Science and Biotechnology, Faculty of Chemistry, Brno University of Technology, Brno 612 00, Czech Republic; matouskova@fch.vut.cz (P.M.); marova@fch.vutbr.cz (I.M.)

**Keywords:** P-3-HB, PHB, wet spinning, microfiber, pores

## Abstract

In this study, we described the development of a simplified wet spinning method of the production of a novel type of porous continuous fiber based on poly-3-(R)-hydroxybutyrate (PHB). The principle of this method is precipitation of PHB dissolved in chloroform solution into the ethanol precipitation bath. The influence of various PHB concentrations and feed rates on specific surface area (measured by nitrogen absorption method) was studied. Materials were also characterized by SEM. Surface areas of fibers achieved by wet spinning were in the range of tens of m^2^.g^−1^, and the biggest surface area value was 55 m^2^.g^–1^. The average diameter of fibers was in the range of 20–120 μm and was dependent on both PHB concentration and feed rate. Optimum conditions for reaching stable fibers of high surface area were 3–5 % w.t. of PHB and feed rate 0.5–3 ml.h^−1^. Fibers were functionalized by adsorption of some natural plant extracts. The incorporation of active substances into fibers was confirmed by infrared spectroscopy. High antioxidant and antimicrobial effect of PHB-fibers with cloves extract was found, as well as excellent long-term stability and optimal dynamics of the release of active compounds. The newly produced material would be applicable in pharmacy, cosmetics, and wound healing.

## 1. Introduction

Polyhydroxybutyrate (PHB) from a family of polyhydroxyalkanoates is a deeply studied polymer with big application potential in material, medicinal, agricultural, and protection fields [1,2,3,4,5]. The main advantage of this well-known stiff and a rather brittle biopolymer of high crystallinity is its production from waste food oil via bacterial transformation [6]. The huge production of waste food oil could lead to a promising future of PHB; however, the feedstock could compete with the needs of biofuel production [7]. Many interesting materials have been prepared based on polyhydroxyalkanoates, especially on PHB. We could find big development in the area of extrusion [8], film production [9], composites [10], scaffolds [11], particles [12], nanofibers [13], conventional fibers [14], filters [15], paper modification [16], cosmetics [17], food packaging [18], and more [19]. Natural hydrophobic properties of PHB could lead towards perspective sorption application [20] of material with high surface area and pores.

All those applications and development are focused on two different groups of PHB processing. The first one works with extruded polymer, while the second one with a dissolved polymer. PHB itself is not easily processed by extrusion because of thermal degradation [21]. Recently, substantial work in this field has been done [5]. However, the processing is not straight forward as in conventional polymers. Due to this problem, an alternative method based on PHB solubilization could be used. More importantly, additional positive properties like higher surface area or porosity could be gained by those methods. For example, solvent casting or wet spinning could be found very useful in the preparation of porous membrane materials [22].

**Figure 1:**
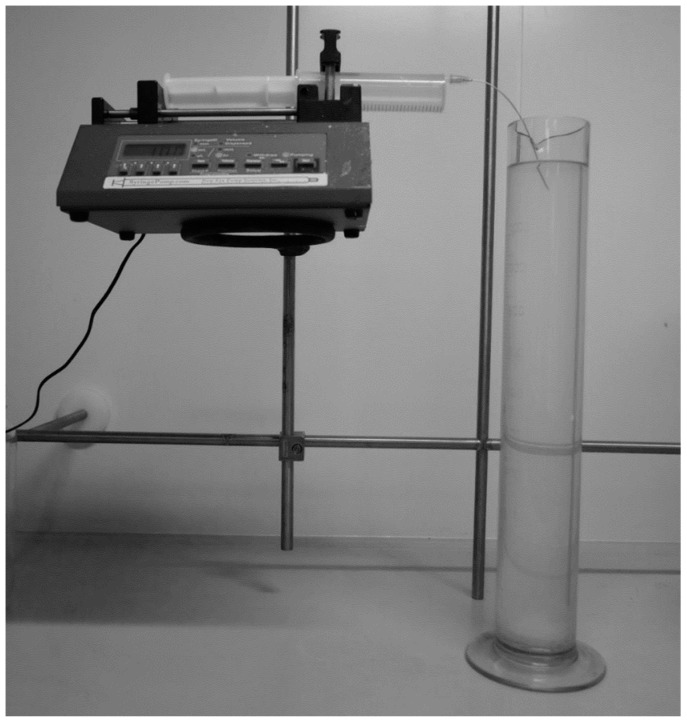
Wet spinning set up.

Wet spinning [23] is one of the oldest spinning techniques originally performed on cellulose spinning from cellulose xanthate [24]. Nowadays, it is mostly used during the production of carbon fibers based on polyacrylonitrile precursor fibers. The general idea of wet spinning is based on continuous precipitation of polymer from an appropriate solution where a solvent is miscible with precipitation bath, and the polymer is not soluble in the bath [23]. For example, precipitation of polyacrylonitrile could be done from a dimethylformamide solution to water. In the first phase, fiber is not yet in the form of the final product. However, a semi-state of protofibril can be reached, which is partly liquid and partly solid and could be drawn, prolonged, and strengthened. Then, it could be dried, lubricated, and winded up. By this method, it is possible to obtain high strength fibers in mono- or multifilament state depending on used spinneret. Wet spinning by its simple set up is possible to practice in laboratory conditions. The simplest set up (Figure 1) could be just from a syringe pump, spinneret, which could be just a simple needle and precipitation bath formed by glassware filled with proper solvent, solution, or a mixture of liquid compounds. The design of experiments could be done by controlling several parameters, where the straightest forward is the speed of the feed-in syringe pump and polymer concentration. Other parameters could be the temperature of bath or polymer solution and concentrations of various additives in solution or precipitation bath. 

In recent literature, the wet spinning process is described with a highly concentrated solution of Poly(3-hydroxybutyrate-co-3-hydroxyhexanoate (PHBHHx) in chloroform [25]. Work is focused on potential in 3D printing of scaffold material and with co-polymer with 3-hydroxyhexanoate units rather than homopolymer PHB.

In our previous study, PHB electrospun meshes were prepared, and levofloxacin was used as the model drug for their functionalization [26]. The effect of the morphology of the electrospun meshes on the levofloxacin release profile was confirmed. Depending upon the morphology, the electrospun meshes released about 14%–20% of levofloxacin during the first 24 h and after 13 days increased up to 32.4%. This amount was relatively low for a potential healing effect.

Based on the above-mentioned data, in this work, a novel type of PHB-based biomaterial with the high surface area using a wet-spinning technique was described. Further, the functionalization of such material by natural antimicrobial compounds was tested to receive new promising biomaterial with optimal and controlled release applicable in many different areas of interest.

## 2. Materials and Methods

### 2.1. Materials

Poly-3-(R)-hydroxybutyrate was obtained from company Nafigate Corp (Prague, Czech Republic). with molecular weights of M_w_ 600 kDa and M_n_ 450 kDa (GPC). Chloroform (pure) and ethanol (96%) were purchased from Penta (Prague, Czech Republic) and used as received. For setting up the spinning equipment, the New Era Scientific syringe pump was used. The syringe was provided by a long needle with a diameter of 0.8 mm and 200 mm length (Figure 1).

### 2.2. Preparation of Fibers by Wet Spinning

PHB was dissolved in heated chloroform during stirring in a closed vial to obtain concentrations in the range of 1–10 wt.% (1, 3, 5, 7.5, and 10 wt.%). Solutions were transferred to the syringe and were pushed out through nozzle to precipitation bath made from ethanol on ambient or cooled temperature. Precipitation bath had to be in a graduated tall cylinder to extend the path of fiber precipitation (Figure 1). Various rates of feed were tested from 0.5 to 15 ml.min^−1^ in various combinations. In cases where fibrous structures were obtained, the material was taken out from the bath, dried on-air, and analyzed by electron microscopy (BET) and nitrogen absorption method (according Brunauer-Emmett-Teller (BET) theory). Some testing material was also analyzed by DSC to obtain the crystallinity of precipitated fiber. 

### 2.3. Characterization of Fibrous Materials

For the characterization of fibrous material, electron microscopy VERSA 3D (FEI Co., Brno, Czech Republic) was used where acceleration voltage was 3 kV, and the regime of detection was set to secondary electrons. All samples were coated with a platinum layer with a thickness of 5 nm. The average diameter of fibers was obtained as an arithmetic mean of ten measurements of thickness on different locations on continuous fiber. In the case of irregular shape (e.g., oval), both maximal and minimal thickness was measured and calculated. Measurement, calculation of average diameter, and the standard deviation were obtained via ImageJ software (1.51, Wayne Rasband, USA). For thermal stability, differential scanning calorimetry was performed using DSC2920 (TA Instruments, New Castle, DE, USA) with a 10 K/min heating regime. Specific surface area screening was determined by the BET method using nitrogen as gaseous adsorbate and performed on the Quantachrome Autosorb-1 porosimeter (Quantachrome, Odelzhausen, Germany). Detailed measurement of pore diameter, specific surface area, and pore size distribution, including adsorption-desorption isotherm, was performed additionally on Quantachrome Nova Station A porosimeter (Quantachrome, Odelzhausen, Germany) using also nitrogen as the gaseous adsorbate. Initial desorption of ambient gases and water vapor removal was done at 100 °C. 

### 2.4. Functionalization of PHB Fibers by Natural Plant Extracts

Four types of plant extracts were used for incorporation into PHB fibers newly prepared by the wet spinning of 5% PHB with a feed rate of 0.5 ml.h^−1^. As model plant materials, water and oil extracts of cinnamon, cloves, oregano, and oak bark were used. Water extracts were prepared by extraction of 1 g of material in 100 ml hot water for 15 min, followed by filtration and centrifugation. Oil extracts were prepared by Folch extraction (chloroform:methanol; 2:1) according to [27], dissolved in chloroform, and stored in darkness at 4 °C. Totals phenolics [28] and antioxidant activity [29] were determined by spectrophotometry. Antimicrobial activity was evaluated according to [26]. Fibers were exposed to water and oil extracts for 24 h at laboratory temperature and darkness. Incorporation of natural extracts into PHB fibers was characterized by FT-IR in attenuated total reflection mode with a single-reflection diamond crystal using a Nicolet iS50 spectrometer (ThermoFisher Scientific, Waltham, MA, USA) according to [26]. 

Plant extracts loaded in PHB fibers (100 mg) were poured in 2 ml of water. The vials were stored at laboratory temperature. Short-term stability was measured by phenolics [28] release from PHB fibers for 5, 10, 20, 30, and 60 min. The release data were presented as the average value with the standard deviation (n = 3). Long-term stability was measured after 2 months of storage of dried functionalized fibers at laboratory temperature in darkness as a change of antioxidant activity [29]. The antimicrobial activity of functionalized PHB fibers prepared by wet spinning was tested against gram-positive bacterium *Micrococcus luteus* CCM 1569, gram-negative bacterium *Serratia marcescens* CCM 8587, and yeast *Candida glabrata* CCM 8270. For the antimicrobial testing, dilution tests were conducted according to [30]. Test microorganisms (150 μL) were cultivated in 96-well microtitration plate in the presence of 10 mg of functionalized fibers in 50 μL of water for 24 h. The absorbance of the wells was measured using ELISA reader (BioTek Instruments GmbH, Bad Friedrichshall, Germany) at 630 nm, before and after 24 h incubation at 37 °C. As a blank, 150 μL of culture with 50 μL of water was used. Each sample was tested in triplicate, and the average value with the standard deviation (n = 3) was presented.

## 3. Results

### 3.1. Wet Spinning

**Figure 2:**
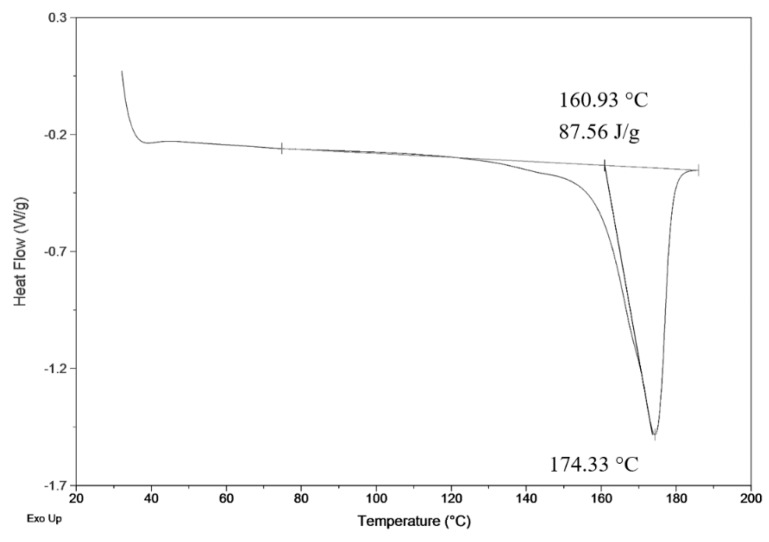
DSC measurement of precipitated fiber (3 wt.%; 3 ml.h^−1^).

Fiber products were characterized by SEM for analysis of morphology differences and by BET to obtain specific surface area. Crystallinity was determined by comparison of measured heat enthalpy 87.56 J.g^−1^ (Figure 2) and reference value 146 J.g^−1^ [26]. By dividing those numbers, we could obtain a resulting crystallinity of 60%, according to Equation (1).

(1) $$X_{DSC}=\frac{\Delta H_{m}}{\Delta H_{m}^{0}}\times 100\%$$

**Figure 3:**
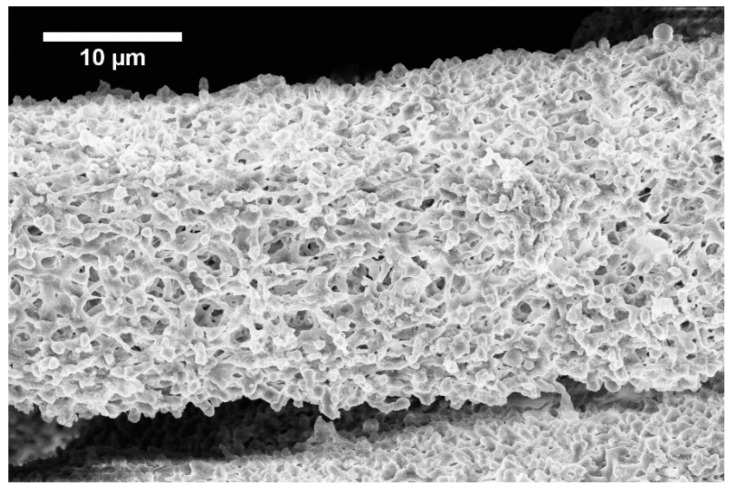
Fiber formed from 1 wt.% solution with 0.5 ml.h^−1^ feed rate.

**Figure 4:**
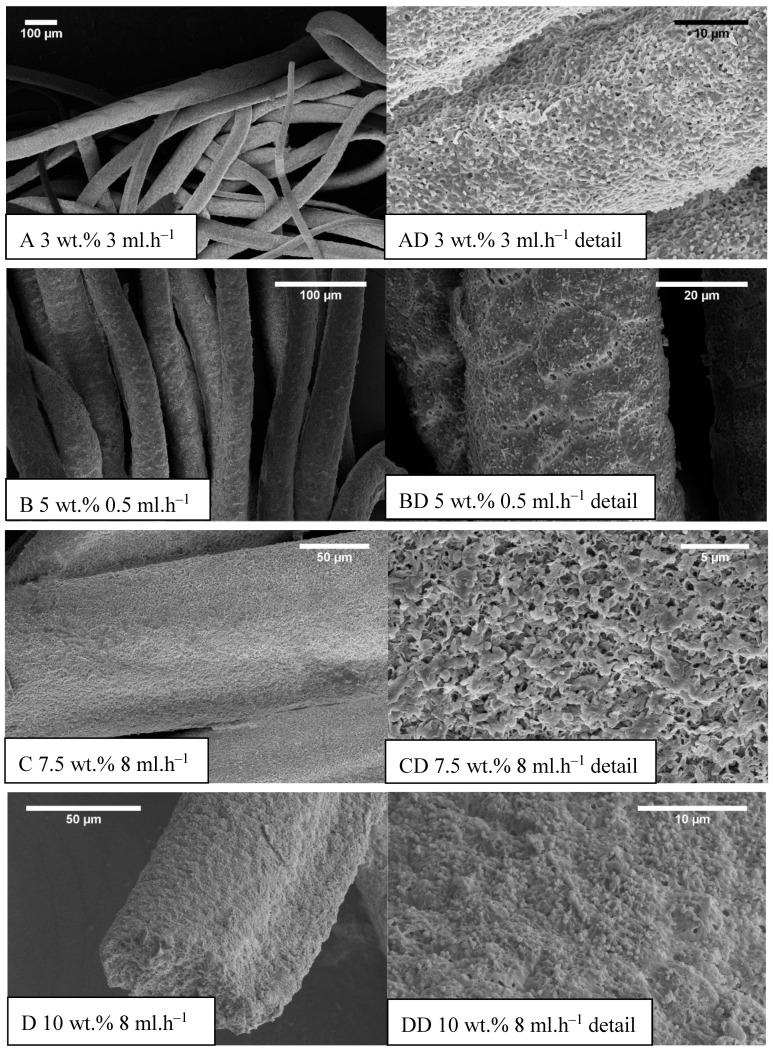
Example SEM images of prepared samples (concentration (wt.%) feed rate (ml.h^−1^)), (**A–D**) the standard magnitude with an overall picture of fibers, (**AD–DD**) detailed measurements.

The development of the wet spinning method started with the idea of the performance of classical wet spinning, where the spun polymer has to be in the form of a stable solution. Polyhydroxybutyrate is soluble in halogenated solvents, where the most used one is chloroform. PHB is insoluble in other common solvents, or thermo-reversible gel is formed. We found that the solution above 10 wt.% was challenging to obtain due to high viscosity and undissolved residues of polymer in the final solution. Therefore, only 10 wt.% and lower concentrated solutions were prepared. For the production needs, it was necessary to achieve the maximum rate in the fiber-forming process. Due to that, the influence of PHB concentration and feed rate of the solution on the precipitation bath were studied. In the case of spinning of 1 wt.% solution at a feed rate of 0.5 ml/h, no fiber precipitation was observed (Table 1). The stream of the solution was just diluted, and the white dust of particles was formed. With a feed speed of 1 ml.h^−1^, the stream of the solution was steady enough to form protofibril, which precipitated continuous fiber (Figure 3). Higher speed, however, did not result in the formation of protofibril, and the stream was decomposed to small fibrils and beads, which were not collected. More useful concentrations for the fiber-forming process started with 3 wt.%. A gradual increase of specific surface was possible to observe also with feeding speed up to 3 ml.h^−1^ (Figure 4A,AD). Faster speed resulted in fibrils and beads, as in the previous case. A higher concentration of polymer in the solution formed a viscous liquid that was capable of spun from the lowest speed rate, which resulted in the highest formed specific surface area of 55 m^2^.g^−1^ (Table 1, Figure 4B,BD). It was challenging to observe specific trends in measured surface areas, depending on the chosen variables of the process. In all measured cases, we observed surface areas in the region of tens of square meters per gram. A higher concentration of PHB and higher speed led to the production of fibers with a very porous surface (Figure 4C,CD); however, the specific surface was lower than in previous cases. The most concentrated PHB solution led to the production of fiber only at higher feed rate due to clogging of needle and precipitation of polymer in the form of a bulky amorphous object on the tip of the needle. However, when faster feed rate was set, a continuous stream of the solution was formed, and during the path in ethanol bath fiber, precipitation was observed (Figure 4D,DD). 

**Figure 5:**
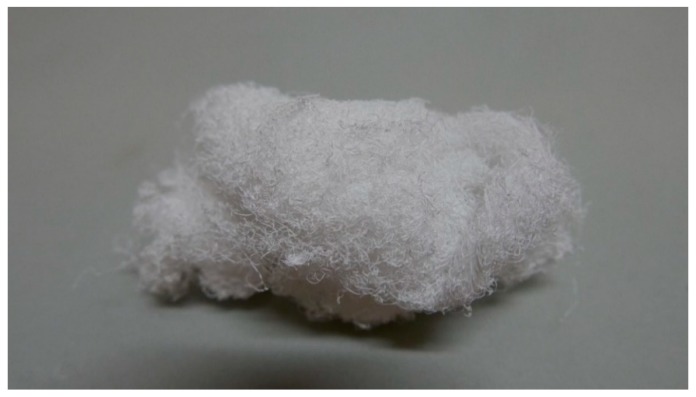
Photography of sample 3 wt.% and 3 ml.h^−1^ detail.

Obtained specific surface areas (Table 1) should be taken as screening results due to the smaller amount of prepared material. Approximately 10 milligrams were analyzed, which could lead to a higher error of the obtained results. Faster feeding didn’t result in fibrous morphologies, especially in low concentrated PHB solutions. Highly concentrated solutions formed fibers only with faster feed speed because of the high viscosity of those solutions, causing the formation of big precipitated lump on the tip of the needle. When optimum velocity set up on the pump was used, the stream of the solution was released and felt down, and a long protofibril was formed, which precipitated around 15–20 cm far from the tip of the needle. By summarizing the results of the study, it was possible to say that for the production of material with a higher specific surface area, the best conditions were: 5 wt.% and very slow speed of 0.5 ml.h^−1^ (Figure 4B,BD). However, with doubling the PHB concentration and using the speed of 15 ml.h^−1^, it was possible to obtain a satisfying result with a lower surface area, but with a much higher production rate. Based on the described production window, it was clear that with higher concentration, it was necessary to increase the feed rate due to the clogging of the nozzle. However, with an increase in speed, it was observed a prolonged area of protofibril formation before precipitation. Due to that effect, it was not possible to study a higher speed with a 10 wt.% solution because of the setup. Based on this observation, we could assume that a bigger precipitation bath with longer precipitation could lead to possible higher production rates. Photography of example prepared material is shown in Figure 5. 

### 3.2. Characterization of Fibrous Material

The average diameter of fibers had an increasing trend with speeding up the syringe pump in most of the cases except 10 wt.% of PHB, where increased feeding speed resulted in a decrease in average fiber diameter (Table 2). It was most probably due to the higher viscosity of the sample and also the higher mass of the initial solution droplet, which goes faster towards the bottom of the precipitation bath. In most cases, fibrous morphology was not regular round but the oval (e.g., Figure 4D). In such cases, the average diameter was measured from two axes, and, therefore, the standard deviation was bigger.

The values of surface area were in the range of tens of square meters per gram of fibers in most measured cases.

**Figure 6:**
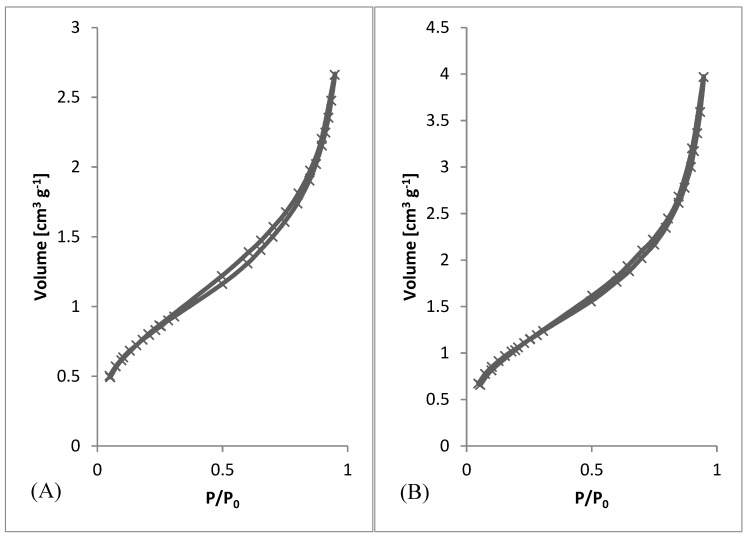

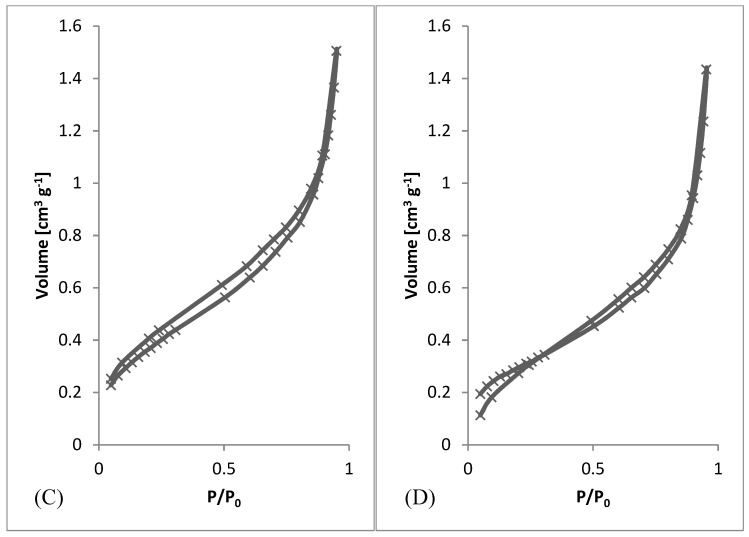
Adsorption/desorption Isotherms. (**A**) 3 wt.%; 3 ml.h^−1^, (**B**) 5 wt.%; 0.5 ml.h^−1^, (**C**) 7.5 wt.%; 8 ml.h^−1^, (**D**) 10 wt.%; 8 ml.h^−1^.

After preliminary screening of specific surface area (Table 1) and obtaining morphology data by SEM (Figure 3 and Figure 4), a higher amount of material was prepared to measure whole adsorption/desorption isotherms (Figure 6). Additionally, to BET measurement, density function theory (DFT) and Barrett, Joyner, and Halenda (BJH) calculations were done to the more precise determination of average pore diameter and pore size distribution. Results of measured surface area, average pore diameter, and total pore volume at selected conditions are introduced in Table 3. Measurements were performed using two types of equipment. The first one (Quantachrome Autosorb-1 porosimeter; Quantachrome, GER) was the same as in the previous screening of BET surface area measurement (Table 1). The second one (Quantachrome Nova Station A porosimeter; Quantachrome, GER) provided also additional BJH and DFT results (Table 3). From those results, we could see some differences between the two measured data sets. Therefore, the method of production could be considered as less reproducible regarding the obtained surface area. However, still, the reasonable surface area was achieved even in large samples. 

**Figure 7:**
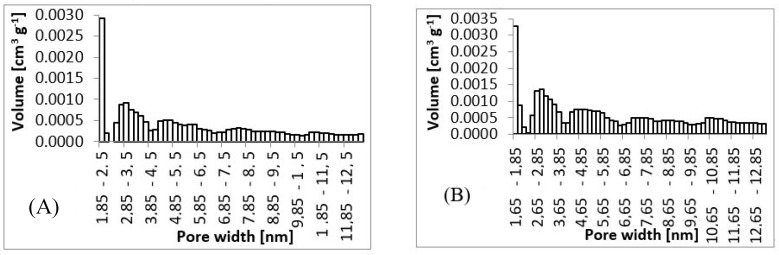

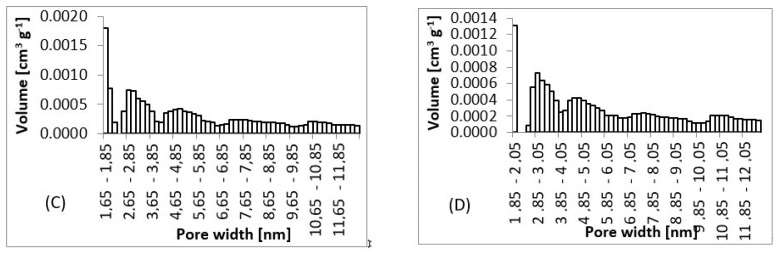
Pore size histograms. (**A**) 3 wt.%; 3 ml.h^−1^, (**B**) 5 wt.%; 0.5 ml.h^−1^, (**C**) 7.5 wt.%; 8 ml.h^−1^, (**D**) 10 wt.%; 8 ml.h^−1^.

From SEM micrographs (Figure 3 and Figure 4), it is visible that pores of obtained material were in the range of sub-micro- to micrometer scale. However, more detailed BET and DFT analysis showed that surface areas formed by nanopores were also present (Figure 7). From the shape of isotherms, we could estimate that the adsorption/desorption mechanism was mostly responsible for the isotherm type III, which corresponded to mesoporous materials (Figure 6). By combining this information from gas adsorption measurement and SEM analysis, we could say that novel material had both characteristics of mesoporous (2–50 nm, according to DFT and BET)/microporous (<2 nm, according to BJH, DFT, histograms) and macroporous (>50 nm, according to SEM) material families.

### 3.3. Functionalization of Fibrous Material by Natural Extracts

As model natural antimicrobial substances, extracts from cloves, cinnamon, oregano, and oak bark were prepared and tested according to Section 2.4. Total phenolic and antioxidant activity of these extracts were analyzed to characterize their potential biological activity. The data obtained are introduced in Table 4.

Data introduced in Table 4 indicate that used plant extracts contained a high amount of total phenolic compounds. Some of them were better soluble in water, the other in oil extracts. Antioxidant activity of both water and oil extracts was also high in most of the samples, especially in water extracts. No direct correlation between phenolics content and antioxidant activity was found. The absorption efficiency of oil extracts incubated for 24 h with PHB fibers (see 2.4) was in the range of 32%–95%, and the highest adsorption activity to PHB fibers was found in cloves extract.

**Figure 8:**
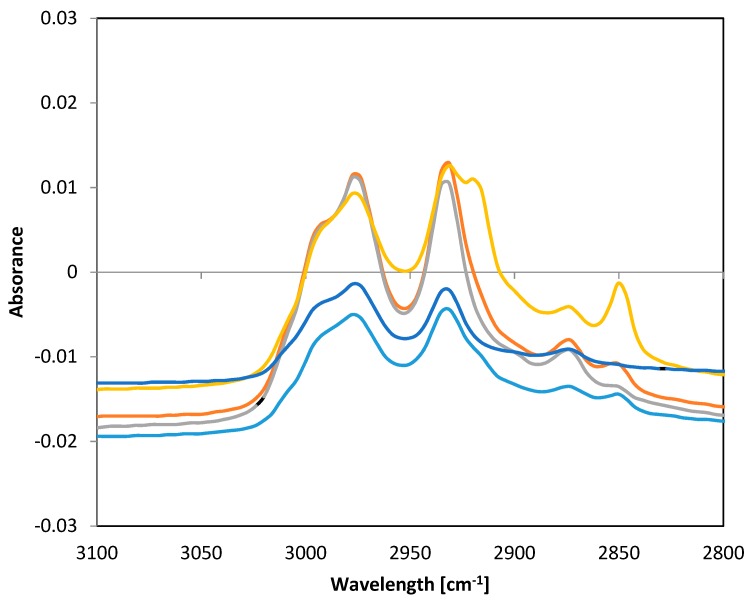
FTIR-ATR spectra of PHB fibers with incorporated plant oil extracts. Note: blue—pure PHB fibers, turquoise—PHB + oak bark, red—PHB + cinnamon, green—PHB + cloves, violet—PHB + oregano.

Fourier transform infrared spectroscopic method with attenuated total reflectance (FTIR-ATR) technique was used for verification that plant extracts were incorporated into the structure of PHB fibers. Absorbance was measured in the frequency range 4000–500 cm^−1^ with a resolution of 8 cm^−1^. Significant differences between individual samples of functionalized fibers were found in the range of 3100–2800 cm^−1^. Spectra of all samples are presented in Figure 8. 

The frequency range of 4000–2500 cm^−1^ corresponded to stretching vibrations of hydrogen bonds in organic compounds, especially C.H, O-H, and N-H. The frequency range of 3300–2700 cm^−1^ could be assigned to C-H bonds. The band 2850 cm^−1^ corresponded to C-H stretching in aldehyde groups [31]. Lipid extracts isolated from natural sources are complex mixtures of different compounds, such as alcohols, ethers, ketones, aldehydes, esters, amines, phenols, etc. [32]. The differences in the spectra of the fibers alone and fibers with extracts showed that some active substances were bound to PHB fibers. The differences in the peak 2850 cm^−1^ indicated that some aldehydes from oil extracts were incorporated into PHB fibers.

Functionalized PHB fibers were stored for evaluation of a long-time (2 months) stability. Samples were stored in dried form according to Section 2.4. Dried samples were then solubilized in water and water:oil emulsion. After 2 months, only marginal changes in antioxidant activity were found no higher than 4%–8% of the original value measured in freshly prepared fibers. 

Short-term stability and release of active compounds were evaluated during 60 min in regular intervals (see Section 2.4). Data were evaluated as a percentage of released total phenolics per time interval from two parallel samples. The complete data set is introduced in Table 5. Short-term stability was strongly dependent on plant material type. After 60 min, 25%–91% of phenolics were released to the water environment, and 28%–82 % to the emulsion, which was more acceptable in topical preparations. The relatively rapid release of some extracts was very important for their biological effect. 

All tested functionalized fibers with adsorbed water, as well as oil extracts, exhibited distinctive antimicrobial effects. Inhibition of microbial growth was observed in G+ and G− bacteria (G+/− for Gramm positive/negative). Moreover, the antimycotic effect against test yeast strain *Candida glabrata* was found, especially in cinnamon and cloves extracts (Table 6). The intensity was dependent on the plant material type and extraction agent. The highest antibacterial effect against both G+ and G− bacteria was found in PHB material with cloves extract. This extract exhibited also the highest antimycotic effect. It should be highlighted that especially oil extracts of all tested plants acted as antibacterial agents with a broad spectrum of activity. Interestingly, the PHB fibers with oil extracts displayed similar efficiency against the gram-negative bacteria compared to gram-positive bacteria. Therefore, these PHB fibers with plant extracts seemed to be highly promising for tissue engineering, medical, and pharmaceutical applications. 

## 4. Discussion 

A series of fibrous materials were prepared by simplified wet spinning from chloroform solution of poly-3-(R)-hydroxybutyrate precipitated through a syringe to ethanol precipitation bath. Materials were collected and analyzed by SEM and BET. First, preliminary BET analysis was performed, obtaining results for specific surface area. Using this screening together with electron microscopy analysis, the average diameter of fibers was measured. According to those results, several conditions were selected to prepare a large quantity of desired material and studied it further by nitrogen absorption-desorption porosimetry and also again by electron microscopy. By preparing a larger amount of materials, it was possible to measure whole absorption-desorption curves and distribution of pores. For the overall information on prepared materials, photography was taken to show wool-like morphology.

Wet spinning [23] belongs to traditional spinning techniques. Nowadays, it is mostly used during the production of carbon fibers based on polyacrylonitrile precursor fibers. Wet spinning is based on continuous precipitation of polymer from an appropriate solution where a solvent is miscible with precipitation bath, and the polymer is not soluble in the bath [23]. Pores of newly obtained material are in the range of sub-micro- to micrometer scale; however, surface areas formed by nanopores are also present. Adsorption/desorption mechanism is mostly responsible for the isotherm type III, which corresponds to mesoporous materials. By combining this information from gas adsorption measurement and SEM analysis, we could say that novel material had both characteristics of mesoporous/microporous and macroporous material families. The high specific surface area of newly produced fibrous material is an important characteristic of future applications. As the optimum conditions for fibers with a higher specific surface, 5 wt.% of PHB and speed of 0.5 ml.h^−1^ were found. These conditions were used for the preparation of a high amount of fibrous material for testing of functionalization and biological properties.

Natural antimicrobial substances, especially phenolic compounds, can be used as stand-alone or adjunctive therapies with antibiotics. The use of essential oils or other plant extracts may exert synergistic antimicrobial activity [31]. However, there are certain limitations, such as low water solubility and low stability. Adsorption of natural antimicrobials to biodegradable nanofibers might help to increase these components’ chemical stability, and solubility also supports controlled and sustained release, enhancing the bioavailability and efficiency against pathogens [32]. So, the antimicrobial efficiency against microorganisms was tested using newly prepared PHA (polyhydroxyalkanoates) nanofibers functionalized by some natural antimicrobials.

All tested PHB fibers with plant extracts showed distinctive antimicrobial activity against the gram-positive bacterium (*M. luteus*), gram-negative bacteria (*S. marcescens*), and the yeast *Candida glabrata*. The antimicrobial efficiency corresponded with the type of entrapped extract. The dynamics of the release of active compounds is probably strongly related to the morphology of the newly prepared PHB fibers [33]. In comparison with our previous study, the release of the antimicrobial drug from electrospun meshes was substantially slower [26]. It should be highlighted that a high specific surface area was probably responsible for a relatively high degree of adsorption (namely in cloves extract) and also for the short-term release of active compounds (50%–90% during 20–60 min) into an environment similar to the skin surface (emulsion). Together with the excellent long-term stability of functionalized PHB fibers, this material seemed to be highly promising for skin covering, wound healing, and supporting therapy of bacterial and fungal infections [34]. Another application of such fibrous material could be in food packaging as antimicrobial adsorption material.

## 5. Conclusions

In this work, a novel porous material with a relatively high specific surface area was prepared by a simple and versatile method of gravity-driven wet spinning of a chloroform solution of PHB into a precipitation bath of ethanol. Such type of material based on PHB was not described yet. The described method of preparation could be possibly enhanced and extended towards multifilament production, which can find application as a material for sorption or drug release. Obtained fibers exhibited diameter in the range of tens of micrometers and internal pores in the range covering macro- to a microscopic scale. Obtained specific surface areas were in the range of tens of m^2^.g^−1^. Application potential was verified by the functionalization of newly prepared fibers by antimicrobial natural extracts. Significant antibacterial and antimycotic effects were found in all types of functionalized fibers, especially in oil extract from cloves. Excellent long-term stability and controlled release of active compounds in the optimum period were probably dependent on specific morphology and high skin surface area of new PHB fibers prepared by wet spinning. The properties of produced novel porous material could predict its broad use as scaffold or carrier for active compounds, such as drugs, enzymes, and additives in pharmacy, cosmetics, and wound healing applications.

## Figures and Tables

**Table 1:** Screening of specific surface areas of prepared samples (BET) [m^2^.g^−1^].

Conc. (wt.%)	1	3	5	7.5	10
Feed (ml.h^−1^)					
0.5	N.A.	23	55	N.A.	N.A.
1	0*	17	36	16	N.A.
2		18	15	15	N.A.
3		43	20	21	29
5			17	23	13
8			16	28	34
10				11	17
15					25

Note: Low (approx. 10 mg) amount of fibrous material was used for analysis. *—material was found nonporous according to BET; however, SEM measurement showed the porous bulk structure. N.A.—the process of precipitation resulted in particles or didn’t form fiber that was possible to collect.

**Table 2:** Average diameter of fiber (μm).

Conc. (wt.%)	1	3	5	7.5	10
Feed (ml.h^−1^)					
0.5	N.A.	41 ± 18	40 ± 6	N.A.	N.A.
1	19 ± 2	40 ± 7	46 ± 10	29 ± 15	N.A.
2		53 ± 6	51 ± 7	56 ± 9	N.A.
3		58 ± 11	63 ± 11	66 ± 11	136 ± 12
5			74 ± 3	69 ± 12	86 ± 10
8			75 ± 22	119 ± 22	72 ± 15
10					72 ± 10
15					83 ± 10

**Table 3:** Detailed analysis of fibrous materials prepared by different conditions.

Sample/Measurement	3 wt.%; 3 ml.h^−1^	5 wt.%; 0.5 ml.h^−1^	7.5 wt.%; 8 ml.h^−1^	10 wt.%; 8 ml.h^−1^
Surface area BET^0^ [m^2^.g^−1^]	43	55	28	34
Surface area BET^1^ [m^2^.g^−1^]	16.1	25.1	15.2	8.5
Surface area BET^2^ [m^2^.g^−1^]	18.5	28	14.7	11.5
Surface area BJH^2^ [m^2^.g^−1^]	25.8	38.4	21.3	22.9
Surface area DFT^2^ [m^2^.g^−1^]	12	18.3	10	7.6
Pore diameter BJH^2^ [nm]	1.4	1.4	1.4	1.4
Pore diameter DFT^2^ [nm]	5.6	6.3	6.5	8.2

^0^—BET analysis by Quantachrome Autosorb-1 porosimeter—screening from Table 1. ^1^—BET analysis by Quantachrome Autosorb-1 porosimeter. ^2^—BET analysis by Quantachrome Nova Station A porosimeter.

**Table 4:** Characteristics of extracts and adsorption capacity to PHB (polyhydroxybutyrate) fibers.

Extract	Phenolics mg/g of Plant Material		ABTS* mg of Trolox/g of Plant Material		Absorption Efficiency % ABTS /100 mg of Fibers
	Water	Oil	Water	Oil	Oil
Cinnamon	7.5 ± 0.8	43.8 ± 1.3	34.2 ± 1.3	13.3 ± 0.8	41.5 ± 2.3
Cloves	11.6 ± 0.8	65.2 ± 2.2	14.1 ± 1.2	6.9 ± 0.6	95.1 ± 5.2
Oregano	37.5 ± 1.7	32.5 ± 1.2	37.5 ± 1.8	14.8 ± 1.1	35.8 ± 1.9
Oak bark	34.1 ± 1.3	11.6 ± 0.7	32.2 ± 0.8	15.3 ± 1.1	32.5 ± 0.9

*—2,2’-azino-bis(3-ethylbenzothiazoline-6-sulfonic acid).

**Table 5:** Short-term stability and release of phenolics from fibers in water and emulsion.

Extract	Water (% of Phenolic Release)					Microemulsion (Phenolic Release %)				
	5	10	20	30	60	5	10	20	30	60
Cinnamon	47.2	62.4	83.5	86.1	91.5	29.3	41	61.9	68.4	82.6
Cloves	34.6	62.9	67.1	71.6	86.8	28.3	54.2	52.7	61.2	71.4
Oregano	21	22.7	27.5	30.4	34.5	-	5.1	9.6	24.7	28.8
Oak bark	7.4	11.5	15.7	15.8	25.8	9.8	16.8	30	40.7	61

Note: Data are expressed as a mean of two parallel analyses.

**Table 6:** Antimicrobial effect of functionalized fibers.

Extract	*C. glabrata* (% of Cell Count Decrease = % of Antimicrobial Effect)		*M. luteus* (% of Cell Count Decrease = % of Antimicrobial Effect)		*S. marcescens* (% of Cell Count Decrease = % of Antimicrobial Effect)	
	Water	Oil	Water	Oil	Water	Oil
Cinnamon	16 ± 0.8	50.2 ± 3.3	8 ± 0.6	73.2 ± 4.6	37.1 ± 1.5	53.5 ± 4.3
Cloves	21.5 ± 1.8	58.5 ± 3.1	47.5 ± 3.1	56.6 ± 3.1	42.9 ± 2.2	62.9 ± 5.6
Oregano	30.8 ± 2.5	4.3 ± 0.8	42.2 ± 3	39.1 ± 1.8	–	32 ± 2.3
Oak bark	35.4 ± 2.8	25.2 ± 1.6	8 ± 0.5	13 ± 0.9	17.1±1.4	43.7 ± 2.2
none		1.2 ± 0.1		3 ± 0.2		2.4 ± 0.1
